# INPATIENT REHABILITATION FOR A PATIENT WITH COVID-19 EXACERBATION OF PULMONARY FIBROSIS: A CASE REPORT

**DOI:** 10.2340/jrm-cc.v8.40698

**Published:** 2025-03-06

**Authors:** Heather MCKENNA, Erin Y. HARMON

**Affiliations:** 1 Cardiopulmonary and Orthopedic Department, Sunnyview Rehabilitation Hospital, Schenectady, NY, USA; 2 James A. Eddy Research Institute, Sunnyview Rehabilitation Hospital. Schenectady, NY, USA

**Keywords:** case reports, COVID-19, idiopathic pulmonary fibrosis, rehabilitation

## Abstract

**Objective:**

To evaluate the benefits of inpatient rehabilitation for a patient with post-COVID-19 pulmonary fibrosis and to provide guidance for rehabilitation professionals, as many conventional therapeutic interventions are not tolerated and are poorly defined.

**Design:**

A case report.

**Subjects/patients:**

A 72-year-old man with a COVID-19-related idiopathic pulmonary fibrosis exacerbation.

**Results:**

The patient was admitted to inpatient rehabilitation with hypoxia and poor endurance for functional activities. Rehabilitation activities were focused on providing patient/family education, energy conservation, low level activities to build strength, problem solving for mobility, and discharge planning within safe medical parameters. Rehabilitation therapies were graded to meet the patient’s physiologic needs and focused on patient and family training. The patient made limited functional gains and continued to have high oxygen needs but achieved his goal of returning home.

**Conclusion:**

Patients with COVID-19-related idiopathic pulmonary fibrosis exacerbations can be treated in acute rehabilitation effectively. With more patients developing post-COVID-19 pulmonary fibrosis, appropriate rehabilitation strategies are important for safe discharge planning. Prioritizing patient/family education may allow these more medically fragile patients to return home.

## LAY ABSTRACT

A 72-year-old man with a history of interstitial lung disease came to our inpatient rehabilitation facility after spending 21 days in the hospital with COVID-19 pneumonia. Upon admission to inpatient rehabilitation, he was on high amounts of oxygen and had very low oxygen readings on a pulse oximeter. He was unable to a transfer, walk, eat, or talk without his oxygen levels dropping, despite using 10 liters of oxygen per minute. The therapy team worked to tailor a program to this patient’s critical needs. Activities were focused on education, low level cardiovascular activities, modifications to activities of daily living, and even household mobility to be able to reach the goal of sending this patient home. This case study describes the program used and demonstrates how inpatient rehabilitation can help patients with an exacerbation of pulmonary fibrosis and pulmonary symptoms after COVID-19.

COVID-19 has claimed the lives of over 1.1 million people in the United States, with over 5.8 million hospitalized (1). Patients with preexisting chronic pulmonary conditions, including interstitial lung disease (ILD), are highly vulnerable to severe disease. ILD comprises multiple pulmonary conditions, defined by diffuse inflammation of interstitium resulting in restrictions of lung tissue and impaired gas exchange (2). Patients with ILD have increased risk of death from COVID-19 compared to adults without ILD after controlling for age, sex, and comorbidities (3, 4).

Idiopathic pulmonary fibrosis (IPF) is a type of ILD of unknown origin with high mortality rates and no cure. IPF diagnosis is based on a series of guidelines and defined features on chest computed tomography (CT) imaging (2). Traction bronchiectasis, widening of the bronchioles due to fibrosis and inflammation, is associated with mortality in both patients with IPF and Post Covid Pulmonary Fibrosis (PCPF) (5–7)*.*

Both reversible and irreversible scarring can result from COVID-19 at all stages of severity (8–10). PCPF sequelae are not fully defined in the literature, as fibrosis resolves in some patients yet persists in others (7, 9). Patients with IPF are at higher risk of lung damage following infection, due to baseline fibrosis. IPF exacerbation with COVID-19 has been documented (11), and research suggests higher mortality rates with COVID-19 among patients with this condition (12).

Inpatient rehabilitation has been shown to benefit patients with IPF (13) but has not been described for patients with IPF and COVID-19. Case reports suggest that patients with IPF following COVID-19 have a progression in their fibrosis (11, 14). This is the first case study describing the rehabilitation strategies for a patient with a COVID-19 IPF exacerbation. Challenges and benefits of inpatient rehabilitation are discussed.

## CASE REPORT

A 72-year-old man was hospitalized with worsening shortness of breath and COVID-19 pneumonia. The patient was vaccinated (with booster). He was treated with Remdesivir and intravenous steroids (dexamethasone) and was not mechanically ventilated.

The patient had a history of heart failure, atrial fibrillation, and sleep apnea. He was being monitored for IPF (Table I). IPF progression had been gradual, and he reported that his pulmonary condition was not affecting his quality of life. He was not on antifibrotics. Before hospitalization, he did not use oxygen during the day and only required 4 liters (L) of oxygen per minute at night. He was independent with walking, activities of daily living (ADLs), and driving. He was working at home and had just purchased a farm for retirement. In the week before admission, the patient was experiencing more hypoxic episodes and dyspnea. He reported oxygen desaturation into the 70s when walking and required 10 min to recover. This was not his usual condition.

**Table I:** Timeline of events

Date	Event
**July 2021**	CT scan of chest finds reticular densities within the visualized mid to lower lungs, predominantly in the periphery, consistent with PF. Appears improved from prior exam, which may represent improvement of the superimposed atelectasis on the prior exam (the acute inflammatory changes). No honeycomb changes appreciated.
**November 2022 (Day 1)**	Patient hospitalized for COVID-19 pneumonia and treated with Remdesivir and intravenous steroids (dexamethasone)
**December 2022 (Day 22)**	Patient admitted to acute rehabilitation. CT scan of chest revealed acute on chronic ILD.
**January 2023 (Day 37)**	CT scan of chest without contrast revealed reticular and bilateral traction bronchiectasis and mild groundglass attenuation. No zone of normal lung interstitium, diffuse fibrosis in lungs, and adjacent pleura. No honeycomb changes are appreciated.
**January 2023 (Day 49)**	Patient discharged home
**February 2023 (Day 77)**	Patient readmitted to hospital for shortness of breath.

CHF: congestive heart failure; CT: computed tomography; ILD: interstitial lung disease; PF: pulmonary fibrosis.

The patient spent 21 days in the hospital for medical management of his pneumonia and received acute care therapy. He was discharged to inpatient rehabilitation (Table I). He was pleasant, cooperative, and motivated during therapy evaluations. His goals were to get better, breathe easier, and go home. He was on 10 L of O_2_ at rest but became hypoxic (into the 80s) when talking or eating. He had a persistent dry cough. His inspiratory capacity was 1583 mL as measured by incentive spirometry. He was unable to perform a stand pivot transfer or walk 10 feet without desaturating into the 70s while on 10 L of O_2_ via high flow nasal cannula. Balance and manual muscle test scores were within functional limits. He completed only 2 min of the 6-min walk test (10 feet), which was discontinued due to oxygen desaturation (81%). The patient scored 3/10 on the BORG scale of perceived exertion and did not report symptoms with hypoxemia, despite having significant dyspnea. The physician’s order was PO_2_ > 90%, limiting activity with therapy and ambulation on the unit with staff.

An Oximizer, with a high lumen diameter and O_2_ reservoir, was trialed to manage the patient’s high oxygen demands but did not improve his oxygen saturation or decrease his O_2_ requirements. A non-rebreather face mask at 15 L minimized hypoxia and allowed for mouth-breathing during activities. This was incorporated in all therapy sessions to minimize desaturation with activity. The patient was educated on the risks of hypoxia and optimal 2-min recovery times. He remained non-ambulatory due to hypoxemia, and traditional inpatient therapies were not tolerated.

After 1 day, the pulmonologist questioned if the patient was appropriate to remain in inpatient rehabilitation. He met with the patient and his family to discuss other alternatives. The patient could not transfer to a subacute facility with O_2_ use > 10 L. Palliative care was discussed. The physician also suggested the patient’s family investigate a double lung transplant. This upset the family and the patient reportedly felt like our staff was giving up on him.

A repeat CT scan was ordered in January 2023 to reevaluate the patient’s ILD (Table I). Scans revealed a progression pulmonary fibrosis and significant scarring, associated with lung “stiffening” limiting both expansion and diffusion capacity. The findings on the CT scan were discussed in interdisciplinary rounds and were incorporated into activity goals and planning for oxygen needs at home. As a result, the goals of inpatient rehabilitation were tailored to accommodate the patient’s medical status and current state of his IPF. His diffusion impairment, high oxygen needs, and limited activity tolerance were barriers in all therapies.

In a family meeting, the pulmonologist explained that COVID-19 pneumonia has a long recovery. CHF and other acute conditions were still being treated with steroids and diuretics. The patient was referred to his primary pulmonologist to inquire about anti-fibrotics. Patient status, expectations for discharge, and barriers for going home were also discussed.

**Fig. 1:**
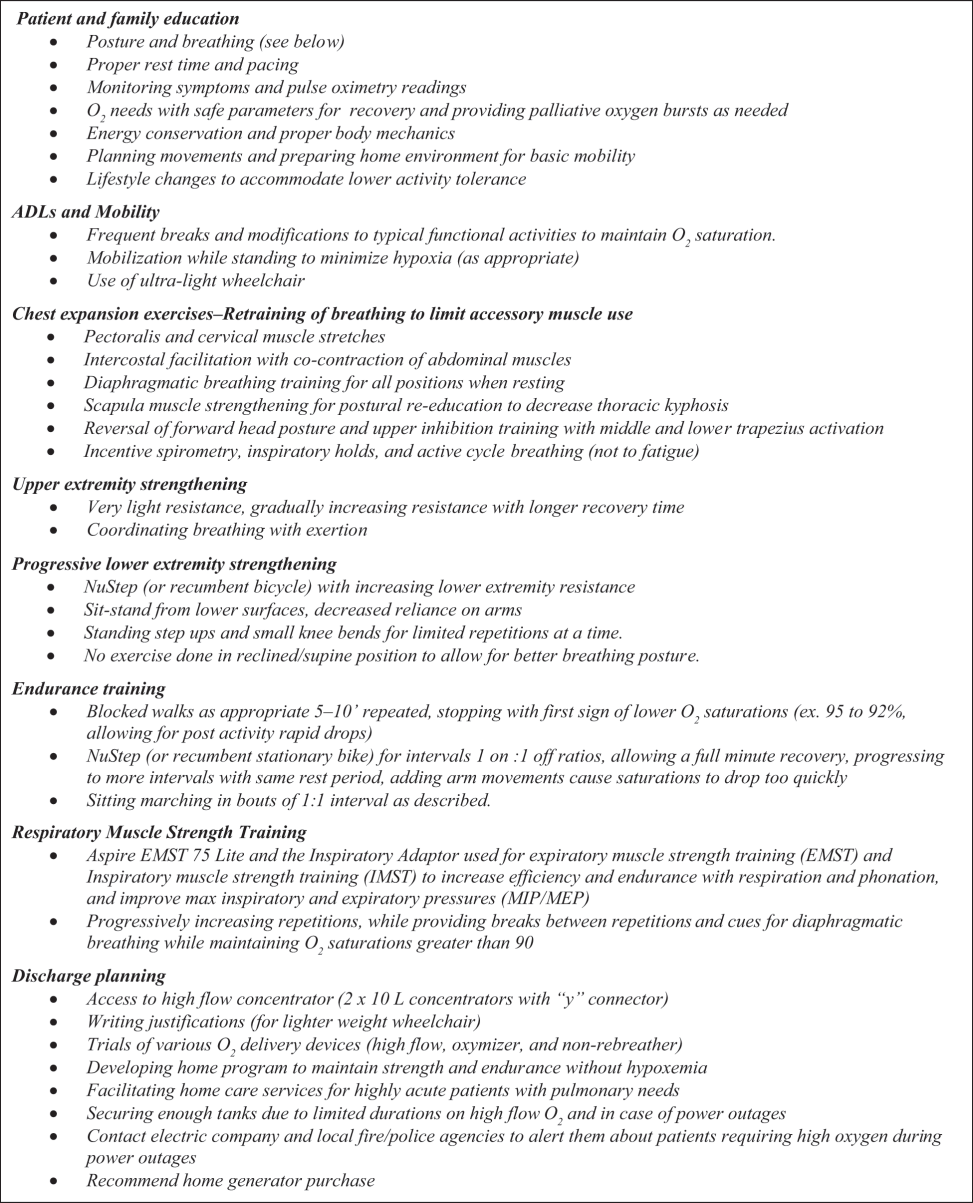
Recommended therapies and considerations for discharge planning. ADL, activities of daily living.

The interdisciplinary team developed a plan to support participation in inpatient rehabilitation. The physician allowed saturations to drop to mid-80s if restored to 88% in a 2-min recovery window, giving the patient more opportunities to participate in therapies. Rehabilitation schedules were coordinated with speech, occupational (OT), and physical therapy (PT). Speech therapy provided respiratory muscle strength training. Activities in OT and PT focused on education, energy conservation, low level activities to build strength, problem solving for mobility, and discharge planning (Fig. 1).

## RESULTS

The patient was in inpatient rehabilitation for 27 days. He demonstrated progress in all areas of self-care and mobility on the Inpatient Rehabilitation Facility Patient Assessment Instrument (IRF-PAI) with the exception of 50’ and 150’ walk and stairs, which he was still unable to complete safely. He was unable to complete the 6-min walk test within safe medical parameters, stopping at 2 min after 15 feet. However, he scored himself 2/10 on the BORG scale of perceived exertion during this activity. This was a 1-point drop from his reported 3/10 score on admission, which is considered a clinically meaningful improvement (15). Inspiratory capacity showed a slight improvement from baseline (1750 mL). Mean inspiratory and expiratory pressures (MIP/MEP) also improved with RMST; MEP exceeded pressures associated with non-productive cough (< 30 cm H_2_O) but both MIP and MEP were still below normative values for age and gender.

An ultralight weight wheelchair minimized exertion with household mobility and allowed for independence. The patient was able move more efficiently and for longer distances in his wheelchair (50 feet on IRF-PAI). He was now able to manage his oxygen tubing and titrate his oxygen safely with mobility and ADL.

Hands on caregiver training including how to monitor O_2_ levels and when to provide bursts of O_2_ to limit hypoxemia was provided (Fig. 1). Because oxygen tanks with a 10–15 L flow rate emptied within 1 h, the patient’s wife learned to read regulators and changed tanks. Practicing made her less anxious about caregiver responsibilities.

Patients are limited to 10 L of oxygen for home use due to concentrator capacities; in this case, the patient’s diagnosis and markers for severe chronic lung disease warranted higher levels. Arrangements were made for the patient to receive 20 L of oxygen for home (2 concentrators with a “y” connector), allowing for bursts of oxygen when levels dropped below 85% on 15 L (Fig. 1). According to the American Thoracic Society, patients with chronic lung disease on palliative oxygen can have short bursts of high flow oxygen (as high as needed) to relieve dyspnea (16). Oxygen delivery methods were also problematic for returning home. Non-rebreather masks have short tubing, limiting mobility. The patient required weaning to tolerate a high flow nasal cannula with extension tubing. His wife was educated on using the non-rebreather mask on the portable O_2_ tank when needed to maintain O_2_ saturations and to provide rescue oxygen with hypoxemia. Arrangements were made with the oxygen supplier to provide enough portable tanks for home and community use. The electric company, local fire, and police agencies were notified in case of power outages, and a home generator was recommended (Fig. 1).

**Fig. 2:**
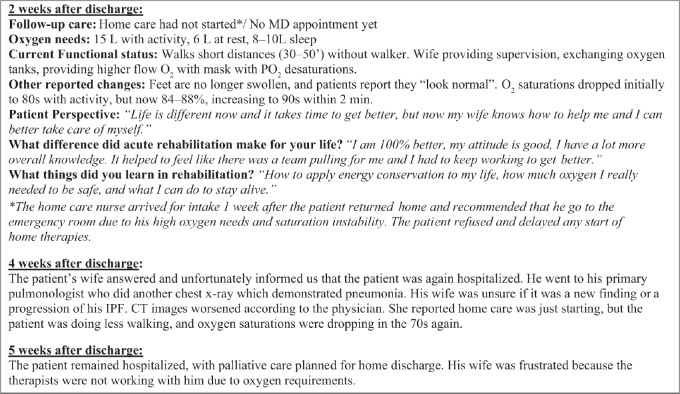
Post discharge outcomes and patient perspective. IPF, Idiopathic pulmonary fibrosis.

While rehabilitation was successful in returning the patient home, functional gains and oxygen titration were limited. The patient and his wife were unable to return to their farm and relocated to a wheelchair accessible apartment near their children and medical facilities. He would be mainly homebound except for medical appointments, but he was happy that he would be safe in his own environment. The patient referred to the rehabilitation staff as “angels” because he felt that even though he had severe symptoms, he could still participate and work with therapists to gain independence and return home. He purchased a NuStep recumbent cross trainer for continued endurance training at home, and home therapy was recommended to maintain his functional gains. The primary therapist followed-up with the patient after discharge to understand his perspective and level of independence sustained (Fig. 2).

## DISCUSSION

This case study highlights the rehabilitation challenges of a patient with IPF exacerbation due to COVID-19. His initial presentation and medical prognosis were poor, but the patient’s positive outlook and rehabilitation course were successful in returning the patient home.

Physicians treat secondary conditions and acute inflammatory changes but have limited medical interventions for patients with IPF. Research has demonstrated the value of inpatient rehabilitation for patients with this condition (13). Fekete et al. emphasized the importance of pulmonary rehabilitation for patients with COPD and COVID-19 (16), but this is the first case study to discuss the challenges of rehabilitation for patients with IPF and COVID-19. As demonstrated in this case report, clinical features made traditional therapies challenging, and in many cases, they were unable to be performed safely due to oxygen requirements. Patients with IPF need direct monitoring of their perceived levels of exertion (BORG scale), O_2_ saturations, heart rate, and blood pressure. Activities should be conducted within safe medical parameters and with sufficient rest between therapies. This case study demonstrates how this can be accomplished for patients with PF and high oxygen needs.

There were multiple tiers of challenges faced. Through interdisciplinary communication, problem solving, family training, and extensive patient education, the patient was able to reach his goal of returning home. Singh and colleagues highlighted the benefits of multidisciplinary collaboration that includes both respiratory and rehabilitation specialists (17).

The patient’s COVID-19 pneumonia, the chronic and progressive nature of his IPF, delays in home care services, and lack of continued home therapies may have impacted the recovery of this patient and were limiting factors in this case report. Individual patient characteristics, medical and functional limitations, and home environment should be taken into consideration when treating patients with similar diagnoses. With a growing population of patients with IPF exposed to COVID-19, it is important to research long-term needs for this population and determine the best ways to deliver rehabilitation services. The complications of sending this patient home suggest improvements are needed in the continuum of care. Patients are being discharged from acute care with higher oxygen requirements and multiple evolving conditions.

In conclusion, discharge planners should consider inpatient rehabilitation facilities as an appropriate discharge destination. Rehabilitation professionals should help patients maintain positive attitudes, train families, find ways promote independence, and change patients’ perspectives on their daily lives. Upon discharge, inpatient rehabilitation therapists should work with all entities of home care services to find ways to prevent rehospitalization. Home care therapists should be prepared to treat patients with higher oxygen needs and evolving pulmonary issues. Collaboration of medical professionals at all levels of care is required to support patients with COVID-19 exacerbated IPF.
